# The optimal exhaled concentration of sevoflurane for intubation without neuromuscular blockade using clinical bolus doses of remifentanil: A randomized controlled trial

**DOI:** 10.1097/MD.0000000000006235

**Published:** 2017-03-03

**Authors:** Eui-Kyoung Goo, Jong Seok Lee, Jae Chul Koh

**Affiliations:** a Department of Anesthesiology and Pain Medicine, Armed Forces Capital Hospital, Bundang-gu, Seongnam-si, Gyeonggi-do; b Department of Anesthesiology and Pain Medicine and Anesthesia and Pain Research Institute, Yonsei University College of Medicine, Seodaemun-gu, Seoul, Republic of Korea.

**Keywords:** airway management, inhalation agent, intubation, neuromuscular blockade, opioid, remifentanil, sevoflurane

## Abstract

**Background:**

The aim of this study was to investigate the optimal exhaled sevoflurane concentration that produces adequate endotracheal intubation conditions when sevoflurane is combined with the different bolus doses of remifentanil used in clinical practice.

**Methods:**

The patients were randomized to 3 groups (groups 1.0, 1.5, and 2.0), receiving remifentanil bolus doses of 1.0, 1.5, and 2.0 μg/kg, respectively. For each group, the concentration of sevoflurane used for each consecutive patient was increased or decreased using the “up-and-down” method based on the success or failure to achieve adequate conditions for intubation in the previous patient. The remifentanil bolus dose was administered 90 s before intubation and after the target sevoflurane concentration was achieved.

**Results:**

In groups 1.0, 1.5, and 2.0, the effective concentration in 50% (EC_50_) of the sevoflurane concentration required to perform successful intubation was 3.0, 2.0, and 1.29 vol% and the effective concentration in 95% was 3.45, 2.91, and 1.89 vol%, respectively. When sevoflurane was administered for the induction, the increase in heart rate (HR) of group 1.0 was the highest among the groups. The highest number of adverse events occurred in group 2.0, including vocal cord rigidity, hypotension, and bradycardia.

**Discussion:**

The EC_50_ of the sevoflurane concentration was 3.0, 2.0, and 1.29 vol% when it was combined with a bolus dose of remifentanil of 1.0, 1.5, and 2.0 μg/kg, respectively. Of the 3 different bolus doses of remifentanil, the dose of 1.5 μg/kg was least associated with changes in the HR/mean blood pressure during intubation without increasing adverse effects.

## 1 Introduction

Remarkable development and improvement have been made in the safety and efficacy of neuromuscular blocking agents. Currently, neuromuscular blockade can be performed more safely and with greater comfort when endotracheal intubation or general anesthesia is performed.

However, in circumstances such as surgery of very short duration, a rapid return of spontaneous ventilation is required. In addition, neuromuscular blockade in patients with respiratory failure or neuromuscular transmission disorders can be dangerous when complete reversal fails. In these situations, general anesthesia without using neuromuscular blocking agents can be beneficial. Although the new agent sugammadex may provide efficacy in this situation by rapid elimination of the remnant neuromuscular blockade,^[1]^ the cost and the insufficient number of clinical trials of this new agent may prevent its routine use as well as several reported adverse effects.^[2–4]^ Therefore, several attempts have been made to develop techniques to perform endotracheal intubation without neuromuscular blockade. A number of studies have shown that remifentanil, sevoflurane, or both agents in combination may provide adequate conditions for laryngoscopy and tracheal intubation in this situation.^[5–7]^ However, there is little number of studies which have measured the optimal concentration of sevoflurane for performing intubation without neuromuscular blockade.

When remifentanil is used in combination with sevoflurane, the drugs can act additively or synergistically on several measures.^[8]^ However, to our knowledge, no studies have identified the effective dose of sevoflurane in combination with different clinically used bolus doses of remifentanil to obtain adequate endotracheal intubation conditions without neuromuscular blockade. The aim of this study was to investigate the optimal exhaled sevoflurane concentration that produces adequate conditions for endotracheal intubation without neuromuscular blockade when sevoflurane is combined with the different bolus doses of remifentanil commonly used in clinical practice.

## 2 Methods

This study was approved by the Institutional Review Board of the Armed Forces Medical Command (Seongnam, Korea, approval no. AFMC-15036-IRB-15-007) and registered on clinicaltrials.gov (identifier: NCT02440204). After they provided written informed consent, male patients aged 18 to 30 years with American Society of Anesthesiologists (ASA) physical status I who were scheduled to undergo elective otolaryngological surgery were enrolled in the study. Exclusion criteria were a history of reactive airway disease, smoking, signs predictive of difficult intubation, and a body mass index ≥ 30 or ≤15 kg/m^2^. The patients were randomized to 3 groups (groups 1.0, 1.5, and 2.0), according to a computer-generated randomization table, to receive remifentanil bolus doses of 1.0, 1.5, or 2.0 μg/kg, respectively.

Two anesthesiologists participated in the induction procedure. One, who was not blinded to the patient's group or target exhaled sevoflurane concentration, recorded the data and adjusted the dose of sevoflurane and remifentanil. The other anesthesiologist, who was blinded, performed the mask ventilation and endotracheal intubation. Patients were premedicated with glycopyrrolate 0.2 mg intravenously. An 18-gauge intravenous catheter was inserted and 0.9% normal saline was infused. In the operating room, all patients were monitored with electrocardiography, pulse oximetry, noninvasive blood pressure measurement, and both inspired and end-tidal concentrations of oxygen, carbon dioxide, and sevoflurane were measured. For each patients, we used Gas Man (Version 4.2; Med Man Simulations Inc, Chestnut Hill, MA) software for simulation of sevoflurane transmission to the target organ (brain). On multiple simulations using various conditions, we discovered that if we maintained the end-tidal alveolar sevoflurane concentration similar to the target concentration over 350 s, equilibrium of the alveolar and vessel-rich organ (brain) concentrations could be achieved. Therefore, induction sequence was set as below.

After preoxygenation for 3 min, anesthesia was induced using a face mask with an anesthetic circuit (Primus, Dräger Korea Co. Ltd, Seoul, Korea) prefilled with 8% sevoflurane. The fresh gas flow was set at 6 L/min and FiO_2_ was set at 1.0. At first, patients were left to breathe spontaneously. However, when the tidal volume was too small (<500 mL) to provide adequate end-tidal sampling for measurement of expiratory gas concentration or the end-tidal carbon dioxide level was above 40 mm Hg, ventilation was assisted. If the patient's spontaneous ventilation disappeared, mechanical ventilation was started with a tidal volume of 10 mL/kg, and the respiratory rate was adjusted to maintain an end-tidal carbon dioxide level between 35 and 40 mm Hg. When the patient lost consciousness and the end-tidal sevoflurane level was higher than the preselected target concentration, the sevoflurane vaporizer dial was set to 0 until the end-tidal concentration became similar to the preselected target end-tidal sevoflurane concentration. Then, the inspired concentration was adjusted to be within a range of 1.0 to 1.4 times the preselected sevoflurane level to identify a concentration that could maintain the preselected target end-tidal sevoflurane concentration steady for at least 3 min. This steady-state end-tidal sevoflurane concentration was maintained for 1 min. If the steady-state concentration was established in <3 min, the remaining period of the 3 min was added to the steady-state maintenance time. After confirmation of the steady state, the bolus dose of remifentanil according to the preselected group was administered via intravenous line over 60 s to prevent chest wall rigidity. Endotracheal intubation was performed 90 s after the end of the remifentanil bolus administration using a 7.5 mm (internal diameter) reinforced endotracheal tube. If the conditions were not good enough to perform intubation because of limited mouth opening or rigid vocal cords, the anesthesia was deepened by increasing the inspired sevoflurane concentration, and rocuronium 0.6 mg/kg was used to facilitate intubation.

The concentration of sevoflurane used for each patient was determined by the response of the previously tested patient using the modified Dixon up-and-down method.^[9]^ The first patient was tested at an end-tidal sevoflurane concentration of 2.5 vol%, which was previously determined as the concentration required for acceptable intubating conditions.^[7]^ Intubation conditions were scored according to the scoring system described by Helbo-Hansen et al^[10]^ (Table 1). Intubation was defined as successful only when the sum of the scores for each parameter was 4 using this scoring system. When the sum of scores was more than 4, it was considered a failure. If intubation failed, the target concentration of sevoflurane for the next patient was increased by 0.5 vol%. If intubation was successful, it was decreased by 0.5 vol%. The cases were collected until 7 pairs of consecutive up-and-down adjustments of the sevoflurane concentration were achieved.^[5,7]^

**Table 1:**
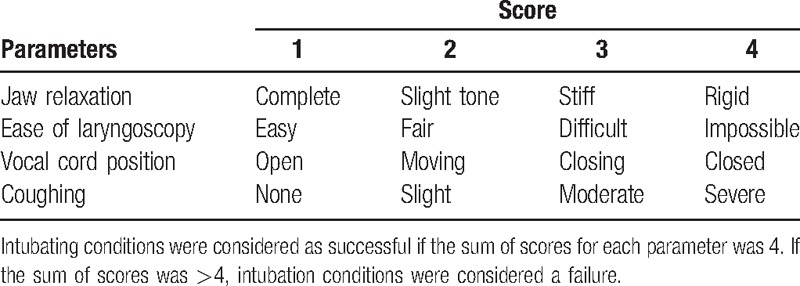
Assessment of intubating conditions.

Mean blood pressure (MBP), heart rate (HR), and oxygen saturation were recorded at initiation, before remifentanil administration, after remifentanil administration, before intubation, after intubation, and 1, 2, and 5 min after intubation. The intubation score and any incidences of adverse effects were also recorded.

### 2.1 Statistical analysis

Statistical analyses were performed using the statistical package R (version 3.2.0) for isotonic regression and SAS (version 9.2; SAS Institute, Cary, NC) for the linear mixed model and analysis of variance (ANOVA). Data are reported as mean ± standard deviation. The sample size was determined to achieve 7 pairs of failure–success to provide the half effective minimum alveolar sevoflurane concentration. To calculate the regression models allowing the prediction of the effective concentration of sevoflurane for successful intubation in 50% (EC_50_) and 95% (EC_95_) of the patients, an isotonic regression using the pooled adjacent violators algorithm (PAVA) was performed for each group. A bootstrapping approach to produce 83% and 95% confidence interval was used for the estimates of EC_50_ and E_95_. ANOVA and a linear mixed model were used to analyze the demographic profiles and hemodynamic data changes in each group. A *P* value <0.05 was considered significant.

## 3 Results

**Figure 1:**
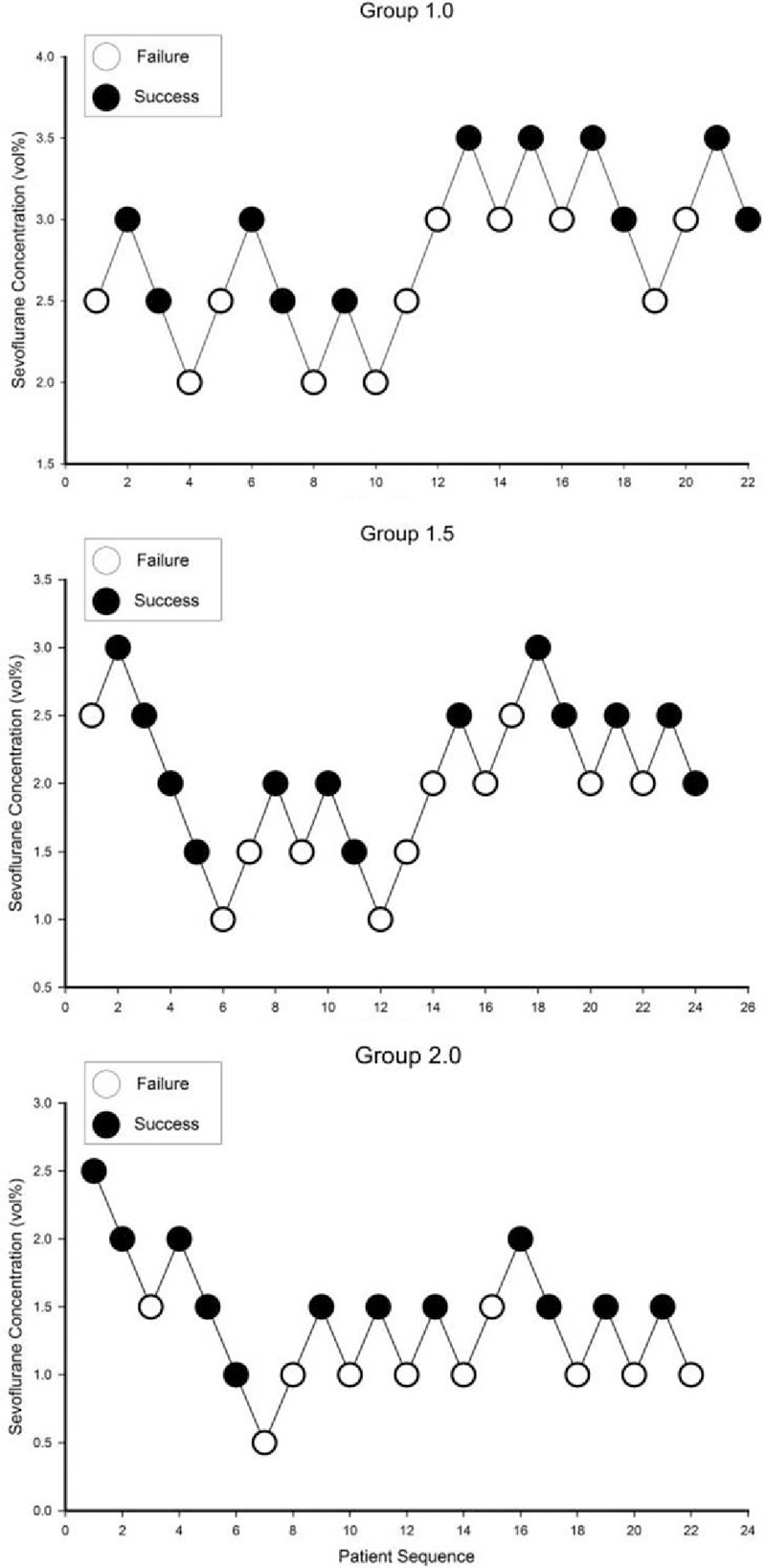
Response crossover plots of the sevoflurane alveolar concentration associated with successful or failed intubation. The starting doses in all groups were 2.5 vol%, and incremental change was 0.5 vol%. Open circles indicate failure of intubation and closed circles indicate successful intubation. 1.0 = remifentanil bolus dose 1.0 μg/kg, 1.5 = remifentanil bolus dose 1.5 μg/kg, 2.0 = remifentanil bolus dose 2.0 μg/kg.

Sixty-eight ASA class I patients participated in this study. To obtain 7 response crossovers in each group, 22, 24, and 22 patients were recruited to groups 1.0, 1.5, and 2.0, respectively. There were no statistically significant differences in the demographics of the patients in each group (Table 2). There were 1, 1, and 3 patients who required neuromuscular blockade for successful tracheal intubation in groups 1.0, 1.5, and 2.0, respectively. The other patients were intubated without neuromuscular blockade. The response crossover plots of the sevoflurane alveolar concentration associated with successful or failed intubation are shown in Fig. 1. By isotonic regression with PAVA and the bootstrap method, there were significant differences in the EC_50_ and EC_95_ of each group (Table 3).

**Table 2:**
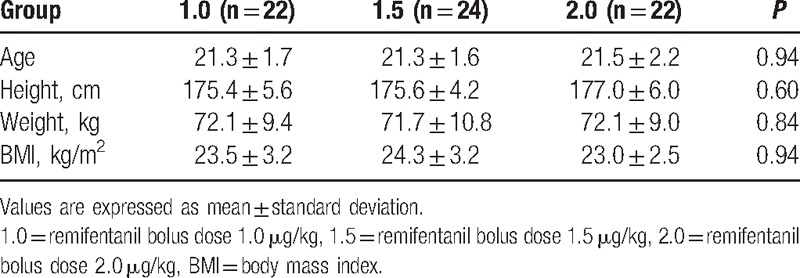
Demographic data of patients in each group.

**Table 3:**
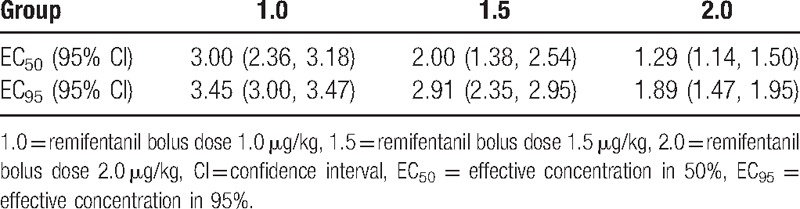
EC_50_ and EC_95_ for successful intubation.

**Figure 2:**
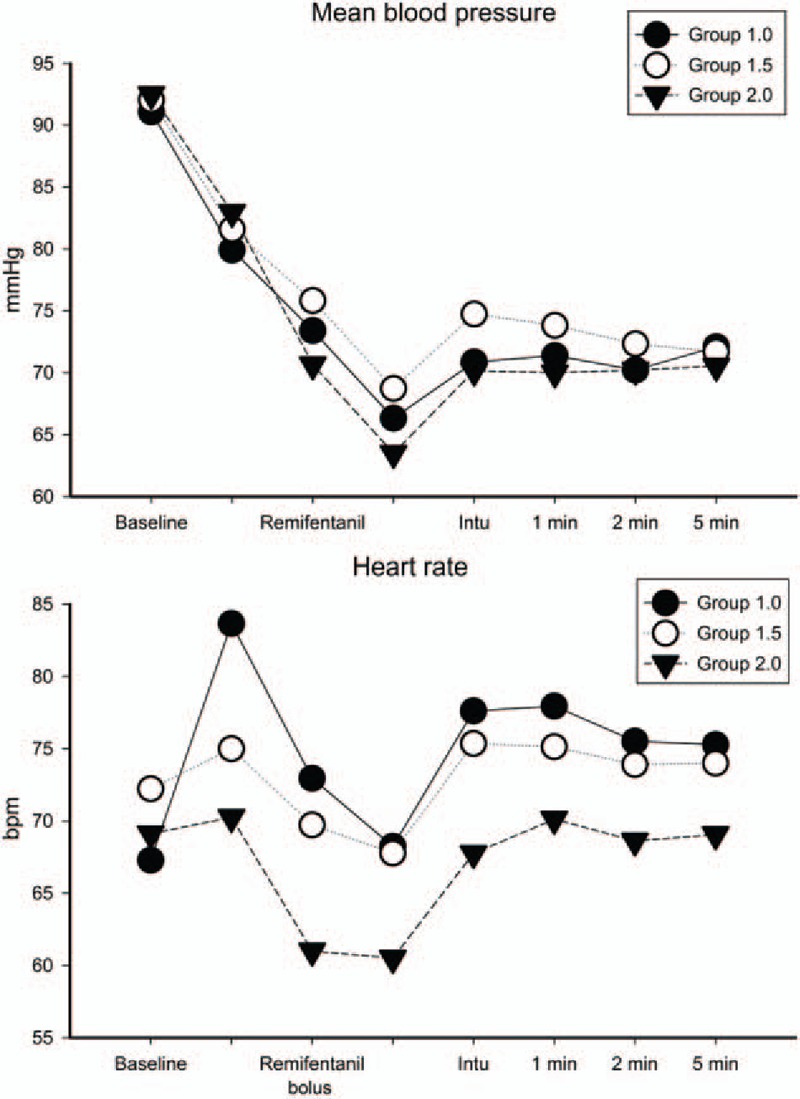
Intraoperative changes in mean blood pressure and heart rate. Closed circles indicate group 1.0, open circles indicate group 1.5, and closed triangles indicate group 2.0. The mean values used in this figure are the calculated least-square means. 1.0 = remifentanil bolus dose 1.0 μg/kg, 1.5 = remifentanil bolus dose 1.5 μg/kg, 1 min = 1 min after intubation performed, 2.0 = remifentanil bolus dose 2.0 μg/kg, 2 min = 2 min after intubation performed, 5 min = 5 min after intubation performed, Intu = intubation performed.

We used a linear mixed model for analysis of HR and MBP. We calculated the estimated least-square means at each time (Fig. 2). There were no significant differences between groups in the initial MBP and HR. In every group, the MBP gradually decreased until intubation and increased when intubation was performed. However, the HR was elevated after induction. When sevoflurane was administered for the induction, the increase in HR of group 1.0 was significantly higher than in the other groups (*P* = 0.014). The HR also decreased when remifentanil was administered and increased after intubation in every group.

There were 3 adverse cardiac events in group 2.0, including 2 patients with bradycardia and 1 patient who had arrhythmia that was successfully managed with 0.5 mg atropine injection. Three patients in group 2.0 required rocuronium injection because of closed vocal cords before intubation. One patient in group 1.5 had bradycardia, and closed vocal cords were encountered in 1 patient in each of groups 1.0 and 1.5.

## 4 Discussion

The results of this study showed that the concentration of sevoflurane required to perform intubation without neuromuscular blockade decreased as the bolus dose of remifentanil increased.

A number of studies have investigated the combination of remifentanil and propofol for performing intubation without neuromuscular blockade.^[6,11,12]^ However, other studies showed that compared with propofol, sevoflurane seems to be a better agent when used as a sole induction agent to perform intubation without neuromuscular blockade.^[13,14]^ Nevertheless, reports of the side effects of using high concentrations of sevoflurane make anesthesiologists hesitant to use it as a sole induction agent.^[15–17]^ In an attempt to lower the required sevoflurane alveolar concentration, intubation without neuromuscular blockade using remifentanil and sevoflurane has been tried.^[5,7,14]^ However, few studies have investigated the effective end-expiratory alveolar concentration of sevoflurane in combination with different doses of remifentanil.

It is more feasible to estimate the concentration of inhalation agents than intravenous agents, by monitoring the exhaled concentration during surgery and anesthesia. Therefore, in clinical settings it would be more useful to adjust the sevoflurane concentration during induction rather than to adjust the dose of remifentanil to achieve effective concentrations of both agents for intubation.

Unlike studies that attempted to identify the effective dose of sevoflurane in combination with different bolus doses of remifentanil,^[7,8]^ this study controlled the participants’ age and general condition within a very narrow range. Only ASA class I patients aged 19 to 28 years participated in this study. Because the required doses of both inhalation and intravenous agents are sensitively affected by the patient's age and general condition, our study provides relatively reliable data because of the tightly controlled age and general condition of the patients.

The results of this study demonstrated that as the bolus dose of remifentanil increased, the minimal required alveolar concentration of sevoflurane to achieve adequate intubation conditions could be decreased. The other studies that have described additive or synergistic effects of the remifentanil–sevoflurane combination support the results of this study.^[7,8]^

We studied 3 different bolus doses of remifentanil in combination with sevoflurane inhalation. Of these 3 doses, 1.0 μg/kg was associated with a requirement for an increase in sevoflurane concentration. According to Cros et al, acceptable intubating conditions were achieved 2 min after using sevoflurane at a concentration of 2.5 ± 0.7 vol% with remifentanil 1 μg/kg injected for 60 s followed by an infusion of 0.25 μg/kg per min. The results of our study showed that the concentration required to perform intubation while using 1 remifentanil 1 μg/kg bolus was higher than this. The absence of the subsequent remifentanil infusion and the relatively young age of the patients in our study might explain these differences.

Unlike the other groups, there was a significant increase in HR after administration of inhalation agents in group 1.0. Sevoflurane has been regarded as an agent that does not cause an increase in HR during induction compared with other inhalation agents.^[18,19]^ However, several reports describe an elevation in HR when using sevoflurane at high concentrations.^[20,21]^ Because the concentration required to perform intubation was highest in group 1.0, the results of this study might support those of previous studies.

By contrast, a remifentanil dose of 2.0 μg/kg was associated with decreased HR and MBP and a lower required dose of sevoflurane. Despite the lowest concentration of sevoflurane being used in group 2.0 in our study, the greatest number of hypotension or bradycardia events was encountered in this group. Several studies have reported the incidence of hypotension and bradycardia after remifentanil use, which may explain our results.^[22,23]^ In addition, there were 3 patients in group 2.0 who had closed vocal cords during the induction period. This seems to be related to the muscle rigidity following a high dose of remifentanil.^[24,25]^ Interestingly, all 3 of the patients who had closed vocal cords had received <1% of the target sevoflurane concentration. Although further studies may be necessary to obtain more reliable data, it seems that sevoflurane may have a role in preventing the remifentanil-induced muscle rigidity because of its own muscle-relaxant property or by other mechanisms.

Compared with the other doses, the bolus dose of 1.5 μg/kg of remifentanil was least associated with HR and MBP changes during the induction period. Changes in MBP or HR during anesthesia can cause an increase in cardiac workload or impaired tissue/organ perfusion. These changes can be devastating to those patients who are vulnerable to these changes. Therefore, it appears that, compared with the other doses, maintenance of alveolar sevoflurane at 2.25 vol% followed by a bolus dose of 1.5 μg/kg remifentanil may provide good intubating conditions without increasing the risk of side effects when we perform general anesthesia without neuromuscular blockade.

In this study, we used Gas Man (Version 4.2; Med Man Simulations Inc) software for simulation of sevoflurane transmission to the target organ (brain). By using this simulation, it took 9 to 12 min to reach steady state between target organ and inspiration concentration when sevoflurane inspiration concentration was kept constant during induction period. However, when steady alveolar concentration was kept constant, it could be lowered within 6 min.^[26]^ In the present study, we have used anesthetic circuit prefilled with 8% sevoflurane in that we could reduce time to reach target alveolar concentration. After we reached target sevoflurane concentration, we maintained the concentration for 390 s (180 s for precise adjustment + 60 s for confirmation, 60 s for remifentanil infusion, and 90 s before intubation). Therefore, we could get sevoflurane concentration similar to the target organ concentration before intubation. During induction with inhalation agent, confirmation of target organ concentration is not very feasible. However, because induction method of this study was designed to achieve target organ concentration as soon as possible, we hope it could contribute to further studies using inhalation agents during induction period.

This study has several limitations. Though we simulated that the brain concentration of sevoflurane could be obtained in 390 s using our method, more accurate brain concentration could be achieved if we were able to maintain the concentration for a longer period of time. However, applying sevoflurane for a longer period of time was not feasible in clinical practice. Furthermore, it was also important to present a feasible model to obtain the target sevoflurane concentration in the brain during the induction period which is easy to apply in actual clinical practice. In addition, because we only included male patients aged 18 to 30 years with ASA physical status I in this study, it is uncertain whether these results could be appropriately generalized to patients of other ages and with comorbidities. However, we believe that result of this study provides good reference data for further studies.

In conclusion, the EC_50_ of the exhaled sevoflurane concentration was 3.0, 2.0, and 1.29 vol% when combined with bolus doses of remifentanil of 1.0, 1.5, and 2.0 μg/kg, respectively. Of the 3 different bolus doses of remifentanil, the bolus dose of 1.5 μg/kg was least related to changes in HR and MBP during intubation without increasing adverse effects.
